# Voxelization algorithms for geospatial applications: Computational methods for voxelating spatial datasets of 3D city models containing 3D surface, curve and point data models

**DOI:** 10.1016/j.mex.2016.01.001

**Published:** 2016-01-13

**Authors:** Pirouz Nourian, Romulo Gonçalves, Sisi Zlatanova, Ken Arroyo Ohori, Anh Vu Vo

**Affiliations:** a TU Delft, Faculty of Architecture and the Built Environment, Department of Architectural Engineering + Technology, Design Informatics, Netherlands; b eScience Researcher at Netherlands eScience Center (NLeSC), Netherlands; c TU Delft, Faculty of Architecture and the Built Environment, Department of Urbanism, 3D Geo Information, Netherlands; d University College Dublin, Environmental Modelling Group, Ireland

**Keywords:** Topological voxelization, Environmental modelling, 3D city models, Point cloud voxelization, Geo-spatial database

## Abstract

Voxel representations have been used for years in scientific computation and medical imaging. The main focus of our research is to provide easy access to methods for making large-scale voxel models of built environment for environmental modelling studies while ensuring they are spatially correct, meaning they correctly represent topological and semantic relations among objects.

In this article, we present algorithms that generate voxels (volumetric pixels) out of point cloud, curve, or surface objects. The algorithms for voxelization of surfaces and curves are a customization of the *topological voxelization* approach [1]; we additionally provide an extension of this method for voxelization of point clouds.

The developed software has the following advantages:

• It provides easy management of connectivity levels in the resulting voxels.
• It is not dependant on any external library except for primitive types and constructs; therefore, it is easy to integrate them in any application.
• One of the algorithms is implemented in C++ and C for platform independence and efficiency.

## Graphical abstract

**Figure:**
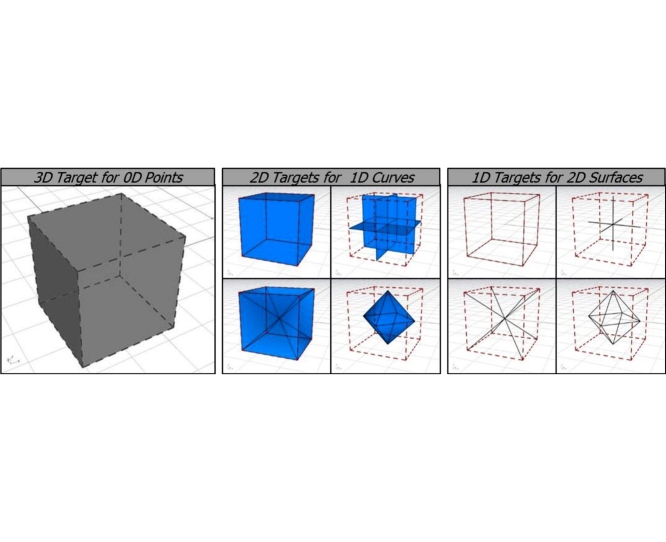

## Method details

### Requirements

• The C++ and C version of our libraries requires the input to be stored in OBJ format, some examples are provided under: https://github.com/NLeSC/geospatial-voxels/tree/master/software/voxelGen/data.
• Rhinoceros3D^®^ and Grasshopper^©^, NURBS Modelling for Windows (Windows 7 or higher) with Dot NET framework (version 3.5 or higher) to run C# version.
• The codes have been tested with PC 64-bit machines with Intel (R) i7 Core(TM) CPU@ 2.50 GHz and installed memory (RAM) of 8 GB.

### Software

• Link: https://github.com/NLeSC/geospatial-voxels/tree/master/software/voxelGen.
• License: Our *software is licensed under the* Apache License Version 2.0 (APLv2).

### Reuse potential

The methods and algorithms can be reused, adapted and integrated with software applications that require voxelization. The installation steps are described in GitHub.

## Introduction

We present three methods for voxelating point, curve, and surface objects. For curve (1D) and surface (2D) objects we present algorithms for the methods mathematically described by Laine [1] and for voxelizing points (0D) we devise our own algorithms. Note that 0D, 1D, and 2D refer to the topological dimension of inputs; this, however does not contradict the fact that all inputs are embedded in three-dimensional Euclidean space of ${\mathbb{R}}^{3}$, i.e. a Cartesian product of X, Y and Z coordinates represented in the domain of real numbers ℝ. Full scripts of algorithms are available in the abovementioned repository. Laine [1] mathematically proves that using the so-called intersection targets desired connectivity levels such as 6 or 26 connected voxel results could be achieved. He does not present an algorithm in detail though. In developing an algorithm for dealing with large datasets, we had to come up with an efficient way of iteration. In general, one can either iterate over all possible voxels in a bounding box or iterate over objects. It sounds obvious that iterating over objects is more efficient as the whole space is not usually filled with objects. This approach can also lead to inefficiency when it comes to a large mesh object such as a TIN (Triangular Irregular Network) terrain model. However, this issue can be resolved if all objects are decomposed into primitives such as triangles (in case of surface input) or line segments (in case of curve inputs). In such cases the algorithms can iterate over triangles or line segments. In our implementation of the topological voxelization method, we have chosen four intersection targets from those defined by Laine, namely two hairline targets for surface inputs and two mesh targets for curve inputs as shown in Table 1. In the following section we give an overview of topological voxelization.

### Topological voxelization in brief

Laine [1] mathematically proves that using the so-called “intersection targets” desired connectivity levels such as 6 or 26 connected voxel results could be achieved. The idea can be best understood by asking the following question based on the definitions of connectivity given below:

• 6-Connected Voxel Collection: a voxel collection in which every voxel has at least one face-neighbour, i.e. by virtue of having adjacent faces.
• 18-Connected Voxel Collection: a voxel collection in which every voxel has at least one edge-neighbour, i.e. by virtue of having adjacent edges.
• 26-Connected Voxel Collection: a voxel collection which every voxel has at least one vertex-neighbour, i.e. by virtue of having adjacent vertices.

**Question 1**: given a curve (1D manifold input, as vector data model) how can we obtain a ‘thin’ voxelated curve (i.e. a voxel collection as a raster representing the curve in question without unnecessary voxels) that is 6 connected or 26 connected?

**Question 2**: given a surface (2D manifold input, as vector data model) how can we obtain a ‘thin’ voxelated surface (i.e. a voxel collection as a raster representing the surface in question without unnecessary voxels) that is 6 connected or 26 connected?

Formally, considering adjacency between voxels in a voxel collection can be in the sense of face-adjacency, edge-adjacency or vertex adjacency. Analogous to these definitions, we can mention edge-adjacency or vertex adjacency between pixels which correspond to 4-neighbourhoods (a.k.a. von Newman neighbourhoods^1^) and 8-neighbourhoods (a.k.a. Moore neighbourhoods^2^) respectively. These neighbourhood definitions are mostly known in definition of Cellular Automata^3^ (CA) models. The term ‘connected’ refers to the graph that represents the adjacencies between voxels or pixels with the given definition of adjacency. It is easier to conceive of the principle of guaranteeing connectivity in raster (pixel or voxel) output by looking at the 2D case.

**1D Input (Curves)**:

• **(saleable) 6-Conncted Intersection Target**: The 6 faces of a voxel cube.
**•** **(saleable) 26-Connected Intersection Target:** 3 plane faces cutting a voxel cube in halves along X,Y and Z, meeting at the centre of the voxel cube.

**2D Input (Surfaces): voxelization**

• **(saleable) 6-Conncted Intersection Target**: The 12 edges of a voxel cube.
**•** **(saleable) 26-Connected Intersection Target:** A 3D crosshair centred at a voxel cube made of lines going through face centres along X,Y and Z axes.

### What is an intersection target?

The fundamental idea behind using Intersection Targets is based on what is called Poincare Duality Theorem [2] similar to the approach of [3], [4]), which establishes a primal-dual pairing between ‘primal’ k-dimensional features and their (n-k)-dimensional ‘duals’ in an n-dimensional space $\left({\mathbb{R}}^{n}\right)$, within which these objects are ‘embedded’. In 3D space, we can think of dualities as shown in Table 4. For a clearer picture we also show potential dual pairs in 1D and 2D spaces respectively in Table 2, Table 3. Simply put, an Intersection Target is a dual feature of dimension n-k (3-k in case of the 3D space of ${\mathbb{R}}^{3}$) that must be intersected with the input feature (primal) to determine whether a voxel should be added to the voxelated output object or not.

A well-known example of duality in 2D maps is the duality between a Delaunay triangulation and a Voronoi tessellation.

An example of dual relationships in 3D is shown in Fig. 2. A face in the left image can be considered as the element through which two 3D cells are connected; this is why representing the same face with an edge in the dual graph makes sense as it connects two vertices representing the respective 3D cells (Fig. 2).

### What is special about topological approach over alternatives?

In many geospatial applications it is important to preserve topological relations among objects [5], [6]. This set of topological relations is used for many purposes such as to analyze relationships between geographical objects [7], to ensure validity of geospatial datasets [8], or to construct ‘graphs’, as in navigation and path planning [9]. In the raster domain, connectivity levels in the output voxels of a voxelation process are essentially topological properties, which are best handled through an explicitly topological approach. The topological approach brings added elegance and clarity to the voxelization process and allows for obtaining desired connectivity levels in a systematic way.

**Fig. 1:**
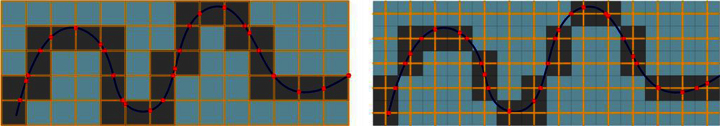
Topological pixilation after [1] the rasterization at the left is based on an ‘outline’ intersection target which ensures a 4-connected rasterized curve in which every pixel has at least one neighbour sharing an edge with; the rasterization at the right is based on a ‘crosshair’ intersection target which ensures an 8-connected rasterized curve in which every pixel has at least one neighbour sharing a vertex with. Resulting pixels are highlighted in dark grey. Intersection Taregts are highlighted in yellow and intersections with them in red dots. Pixels are shown as blue squares.

**Fig. 2:**
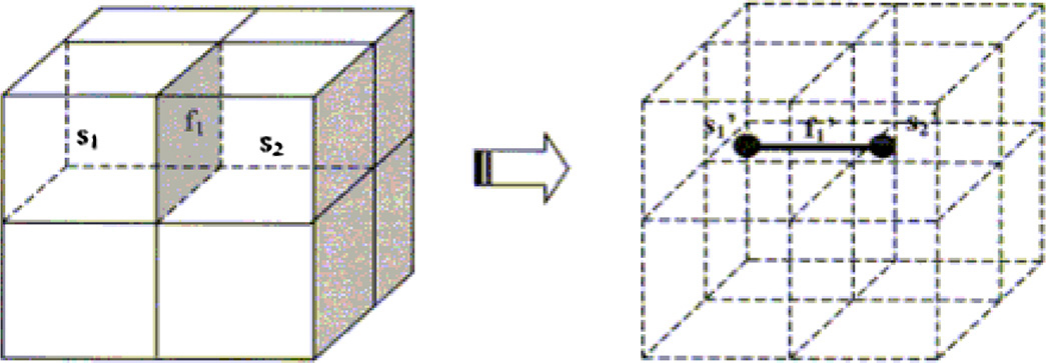
Representing adjacencies between 3D cells or bodies via their dual vertices [4].

In the topological approach to voxelization [9] the idea is that if we are to decide whether a voxel ‘needs to be’ in the output (so as to ensure preserving the topological properties of the input features); we need to ensure its relevant Intersection Target intersects with the input. The nature of these Intersection Targets is dual to the nature of the primal inputs. This way, there will be no doubt on the necessity of having a voxel in the output; i.e. the results are guaranteed to be ‘minimally thin’ as to representing a raster version of the original vector features. We refer the reader to the mathematical proofs given in [1]. Here we give an intuitive explanation for the 2D example shown in Fig. 1. This figure shows a 2D version of topological rasterization (pixelization) of a 1D input curve. If we want to ensure that every necessary pixel is added to the results, we need to focus on the connectivity level desired (4 or 8 neighbours for each pixel). If we want our pixelated curve to be a 4-Connected path so as to best represent the connected underlying curve, then we need a determining factor for including a pixel in the outcome. Let us figuratively imagine an ant going through that curve somewhere, we want to know from which spaces (pixels) it has passed; and that we want to reconstruct its path as a sequence of interconnected pixels sharing a wall with one another. If the ant in question crosses a wall-edge in one pixel it definitely enters to a 4-neighbouring cell. Therefore, checking the crossings of the path curve with the pixel boundaries would be enough to decide for including a pixel in the rasterized path.

The prevalent alternative approaches, which are quite common as to their efficiency can be seen as variants of Scan Conversion [10], which works by interacting rays in 3D directions passing through objects. If all voxels inside are needed then voxels coinciding with intersection points between odd and even intersections will be added to the rasterized (voxelated) output. In our approach this operation corresponds to finding those 0D voxel centre points which happen to be intersecting with the 3D volume (inside or on the volume).^4^ Observe that the last pair of dual features in Table 4 actually corresponds to voxelating a volume in this way.

The topological approach can also eventually be adapted to be implemented based on rays, so as to make it more efficient, but that subject would fall out of scope of the current paper. We can simply say that such a scaling approach would be based on considering Meta Intersection Targets such as rays for meshes and half-planes for curves and finding intersection parameters along these Meta Intersection Targets to locate meeting voxels.

### Point voxelization (0D inputs, 3D targets)

In this section the voxelization algorithm for 0D data inputs, i.e. point clouds, is described in detail. The algorithm has been devised and implemented in C# and its implementation is available in our GitHub repository. Its pseudo code is in Algorithm 1.

In the initialization phase, the algorithm creates a bounding box for ${\mathbb{R}}^{3}$ coordinates that is larger than or equal to the size of the bounding box of the point cloud in ${\mathbb{R}}^{3}$ (1.a, 1.c). It then finds how many voxels could be in each direction by finding the floor integer closest to the size of bounding box in each direction divided by the voxel size in the corresponding direction (1.e, 1.f, 1.g). It then initializes a 3-dimensional array of Boolean (1 bit) values of respective X, Y, and Z sizes plus one (i.e. minimal in size for it only stores bits). This array will be used to keep track of voxels already visited as tuples of [i,j,k] in ${\mathbb{R}}^{3}$. If not yet visited, it marks it as visited (true). It then continues by ‘embedding’ the voxel in ${\mathbb{R}}^{3}$, that is through mapping the [i,j,k] voxel in ${\mathbb{R}}^{3}$ to ${\mathbb{R}}^{3}$ by first creating a point of respective voxel size and then shifting it first for half of the voxel size vector and then for the point at the minimum corner of the ${\mathbb{R}}^{3}$ (2.g,2.h) bounding box.

Algorithm 1 **voxelizePointCloud.**

**Table:** 

**function:** voxelizePointCloud
**input:** Point3d[] PointCloud, *double* sx, *double* sy, *double* sz
**output:***List*<*Point3d*>voxels
1. Initialize
a. form a bounding box BBox in ${\mathbb{R}}^{3}$ for the PointCloud, i.e. composed of six intervals between the MaximumPoint with largest X,Y,Z coordinates and the MinimumPoint with the least;
b. define voxels as new list of 3D points;
c. define voxel size as Vector3d vSize = [sx, sy, sz];
/* generate a bounding box equal or larger than the current bounding box in ${\mathbb{R}}^{3}$ (c.f., Algorithm 2) */
d. ZBBox = **RBBox_to_ZBBox**(RBBox, vSize)
e. compute $CX=\left\lfloor \left(ZBBox.Max.X-ZBBox.Min.X\right)/Sx\right\rfloor$; //number of voxels in X direction
f. compute $CY=\left\lfloor \left(ZBBox.Max.Y-ZBBox.Min.Y\right)/Sy\right\rfloor$; //number of voxels in Y direction
g. compute $CZ=\left\lfloor \left(ZBBox.Max.Z-ZBBox.Min.Z\right)/Sz\right\rfloor$; //number of voxels in Z direction
h. define bool[,,] Voxels3I as a 3D array of Booleans of size [CX,CY,CZ];
i. define List<Point3d>VoxelsOut as new list of 3D points;
2. Compute voxels
a. For each vertex as Point3d in the point cloud do:
i. Compute integer $i=\left\lfloor \left(vertex.X-ZBBox.Min.X\right)/Sx\right\rfloor$;
ii. Compute integer $j=\left\lfloor \left(vertex.Y-ZBBox.Min.Y\right)/Sy\right\rfloor$;
iii. Compute integer $k=\left\lfloor \left(vertex.Z-ZBBox.Min.Z\right)/Sz\right\rfloor$;
/*Check if a voxel was already generated for the {i,j,k} location in ${\mathbb{R}}^{3}$, if not generate one*/
b. If (Voxels3I[i, j, k] = false)
i. $\text{Voxels}3\text{I}\left[\text{i},\text{j},\text{k}\right]=\text{true}$; //mark it as visited
ii. Point3d VoxelOut = new Point3d(i * Sx, j * Sy, k * Sz);
iii. Voxelout = Voxelout + VSize / 2 + Zbbox.Min; // change its reference as to the Z
iv. VoxelsOut.Append(VoxelOut); //and add it to the voxel output
3. return voxels

The ZBBox algorithm adjusts a bounding box in ${\mathbb{R}}^{3}$ to a larger or equal size bounding box in ${\mathbb{R}}^{3}$. Its pseudo code, described in Algorithm 2, uses **MinBoundRP_to_ZP** and **MaxBoundRP_to_ZP** to ensure the new bounding box contains the ${\mathbb{R}}^{3}$ bounding box. The time complexity of the algorithm is linear, i.e., *O*(*n*) where *n* is the number of points in the point cloud.

Algorithm 2 **RBBox_to_ZBBox.**

**Table:** 

**function:** RBBox_to_ZBBox
**input:** A bounding box in ${\mathbb{R}}^{3}$, i.e. composed of three intervals in X, Y, and Z directions
**output:** A bounding box embedded in ${\mathbb{R}}^{3}$, Corresponding to the voxels in ${\mathbb{R}}^{3}$
1. **RBBox_to_ZBBox**:
/*to ensure the bounding box for voxels covers the whole bounding box in ${\mathbb{R}}^{3}$ */
a. Compute Point3d ZMinP = **MinBoundRP_to_ZP**(RBbox.Min, vSize); //min box corner
b. Compute Point3d ZMaxP = **MaxBoundRP_to_ZP**(RBbox.Max, vSize); //max box corner
c. return new BoundingBox(ZMinP, ZMaxP);
2. Function **MinBoundRP_to_ZP(RPoint, VSize)**
a. Compute x = RPoint.X; y = RPoint.Y; z = RPoint.Z;
b. Compute u = VSize.X; v = VSize.Y; w = VSize.Z;
c. Compute $ZP_{x}=u.\left\lceil x/u\right\rceil$; //ceiling integer nearest to the X coordinate of the point in question
d. Compute $ZP_{y}=v.\left\lceil y/v\right\rceil$; //ceiling integer nearest to the Y coordinate of the point in question
e. Compute $ZP_{z}=w.\left\lceil z/w\right\rceil$; //ceiling integer nearest to the Z coordinate of the point in question
f. Return ZP = new $Po\mathrm{int}3d\left(ZP_{x},ZP_{y},ZP_{z}\right)$
3. Function **MaxBoundRP_to_ZP(RPoint, VSize)**
a. Compute x = RPoint.X; y = RPoint.Y; z = RPoint.Z;
b. Compute u = VSize.X; v = VSize.Y; w = VSize.Z;
c. Compute $ZP_{x}=u.\left\lfloor x/u\right\rfloor$; //floor integer nearest to the X coordinate of the point in question
d. Compute $ZP_{y}=v.\left\lfloor y/v\right\rfloor$; //floor integer nearest to the Y coordinate of the point in question
e. Compute $ZP_{z}=w.\left\lfloor z/w\right\rfloor$; //floor integer nearest to the Z coordinate of the point in question
f. Return ZP = new $Po\mathrm{int}3d\left(ZP_{x},ZP_{y},ZP_{z}\right)$

**Fig. 3:**
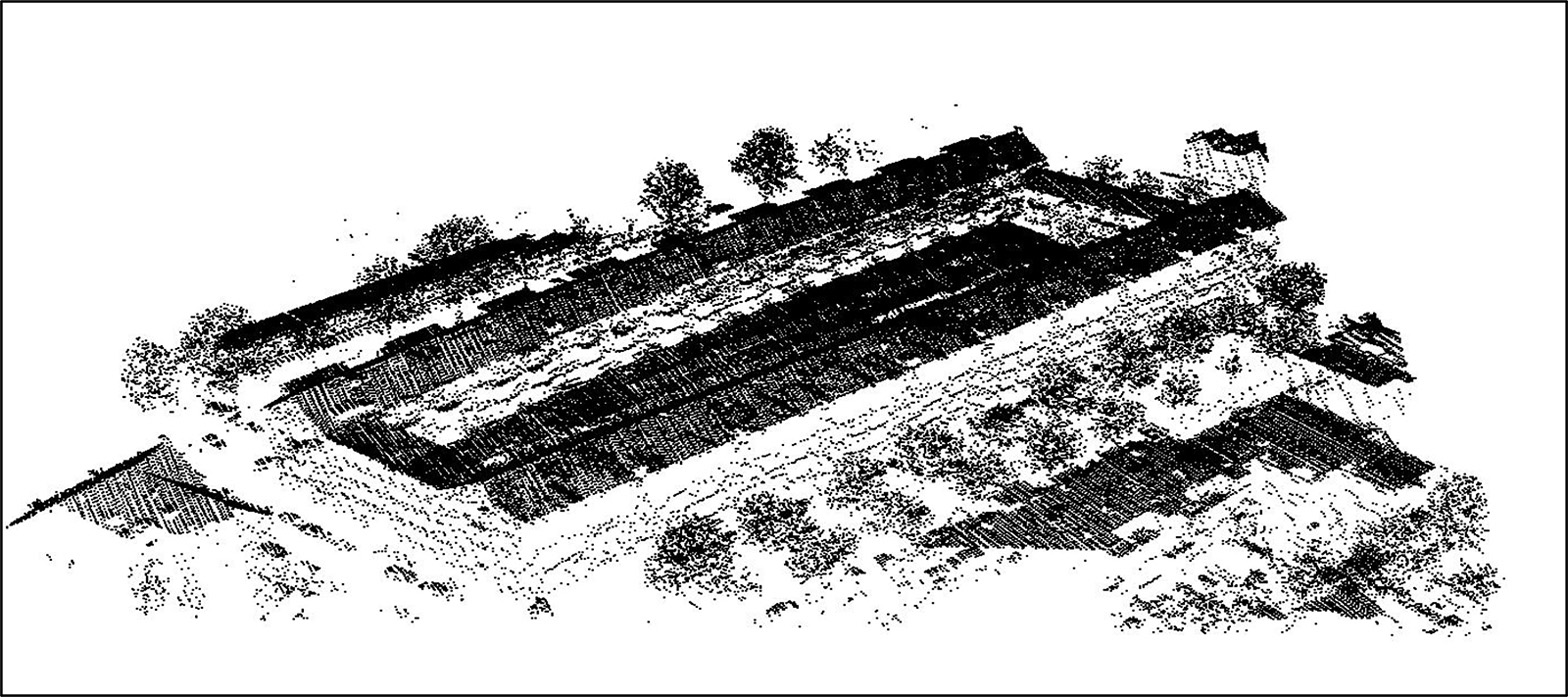
Rotterdam AHN dataset, site in Noordereiland.

**Fig. 4:**
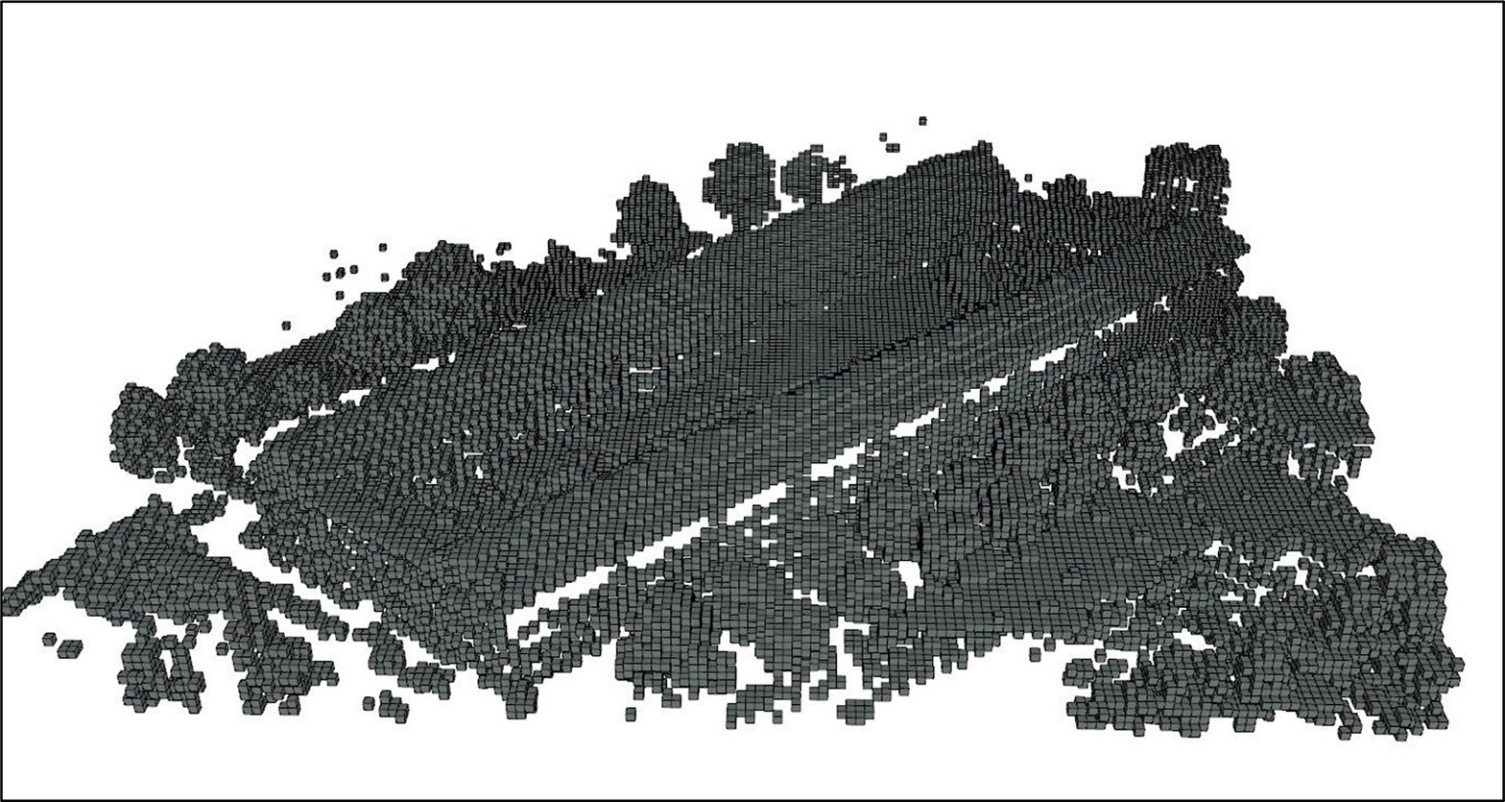
Rotterdam AHN-2 dataset, Noordereiland, voxel count 31,880, 1 m × 1 m × 1 m.

**Fig. 5:**
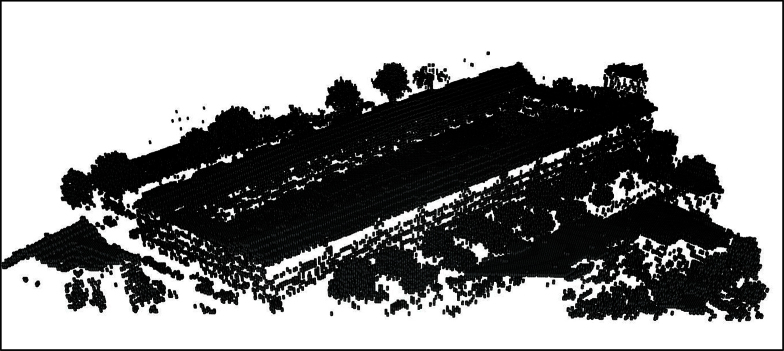
Rotterdam AHN-2 dataset, Noordereiland, voxel count 81,588, 0.4 m × 0.4 m × 1 m.

Fig. 4, Fig. 5 show the application of this algorithm on a sample point cloud data from AHN (Actual Height of Netherlands dataset) of Noordereiland in Rotterdam, shown in Fig. 3. This point cloud is consisted of 136,828 points as X, Y, and Z coordinate tuples, we have provided this point cloud in our GitHub repository.

Fig. 5 depicts voxels with higher resolution than those shown in Fig. 4.

### Curve voxelization (1D inputs, 2D targets)

According to the topological voxelization approach [1] if an effectively one dimensional input such as a curve is intersected with voxels replaced by relevant intersection targets mentioned in Table 1, then the resulting set of voxels is guaranteed to have the desired connectivity level, namely 6 or 26. The idea is to construct intersection targets as triangle meshes and then:

1. form a bounding box for the curve in question;
2. adjust the bounding box to ensure its size is an integer multiple of the voxel size and that it covers the whole curve;
3. fill in the bounding box with voxels; find out voxels which potentially intersect with the curve in question, i.e. those closer than half of the size of a virtual vector representing the diagonal of a voxel cube;
4. for each voxel that is near enough to be possibly included in the result, find out if its relevant connectivity target intersects with the curve in question; if yes then add it to the voxels.

Here we explain our voxelization algorithm for 1D data inputs, i.e. lines, polylines or curves that can be approximated as such.^5^

Algorithm 3 **voxelizeCurve.**

**Table:** 

**function:** voxelizeCurve
**input:** connectivity *int*, curves list<*polyline*>, sx *double*, sy *double*, sz *double*
**output:** voxels *list*<*3D_point*>
1. for each curve in curves:
a. form a bounding box in ${\mathbb{R}}^{3}$ for curve;
b. define voxels as list of 3D points;
/* generate a bounding box equal or larger than the current bounding box in ${\mathbb{R}}^{3}$ (c.f., Algorithm 2) */
c. ZBBox = **RBBox_to_ZBBox**(RBBox, vSize);
d. vSize = [sx, sy, sz];
/* Pseudo code described by Algorithm 6*/
e. VoxelPoints = **BBoxToVoxels**(ZBBox,vSize);
f. /*voxel centre points closer than the length of vSize to the curve which possibly intersect;*/
g. near_voxel_points = VoxelPoints.FindAll(lambda| lambda.DistanceTo(Curve)<|vSize|/2;
f. for each voxel_point in near_voxel_points:
i. if (connectivity = = 26)
/* Pseudo code described by Algorithm 4*/
intersectionTarget = **1D_26_intersectionTarget** (voxel_point, vSize);
ii. elseif (connectivity = = 6)
/* Pseudo code described by Algorithm 5*/
intersectionTarget = **1D_6_intersectionTarget** (voxel_point, vSize);
iii. else
throw exception and prompt: “other connectivity levels undefined”;
iv. if (**TriangleIntersectLine** (intersectionTarget, curve))
voxels.add (voxel_point);
2. return voxels

The idea behnid the algorithm TriangleIntersectsLine is to represent an imaginatry intersection point in the triangle using two barycentric coordinates based on the axes defined along two vectors corresponding to two sides of the triangle. If the barycentric parameters termed s & t can be found and they are both positive and add up to one, then there is an intersection inside the triangle in question. An algorithm for **TriangleIntesectsLine** is available at http://geomalgorithms.com (by *Dan Sunday*, subject: Intersections of Rays, Segments, Planes and Triangles (3D)).

The function 1D_26_intersectionTarge described by the pseudo code in Algorithm 4 creates mesh objects that ensure 26 connectivity if used as intersection target for voxelizing 1D input.

Algorithm 4 **1D_26_intersectionTarget.**

**Table:** 

**function:** 1D_26_intersectionTarge	
**input:** voxel size as Vector3d	
**output:** an intersection target for 1D input ensuring 26 connectivity as a mesh object as in the figure below	
1. Initialize	
a. Mesh IntersectionTarget = new Mesh();	
b. Point3d[] Vertices = new Point3d[12];	
c. double u = (vSize.X / 2), v = (vSize.Y / 2), w = (vSize.Z / 2);	
2. Create Vertices	
a. Vertices[00] = VoxelPoint + new Vector3d(+u, +v, 0);//parallel to XY	
b. Vertices[01] = VoxelPoint + new Vector3d(−u, +v, 0);//parallel to XY	
c. Vertices[02] = VoxelPoint + new Vector3d(−u, −v, 0);//parallel to XY	
d. Vertices[03] = VoxelPoint + new Vector3d(+u, −v, 0);//parallel to XY	
e. Vertices[04] = VoxelPoint + new Vector3d(0, +v, +w);// parallel to YZ	
f. Vertices[05] = VoxelPoint + new Vector3d(0, −v, +w);// parallel to YZ	
g. Vertices[06] = VoxelPoint + new Vector3d(0, −v, −w);// parallel to YZ	
h. Vertices[07] = VoxelPoint + new Vector3d(0, +v, −w);// parallel to YZ	
i. Vertices[08] = VoxelPoint + new Vector3d(+u, 0, +w);// parallel to ZX	
j. Vertices[09] = VoxelPoint + new Vector3d(−u, 0, +w);// parallel to ZX	
k. Vertices[10] = VoxelPoint + new Vector3d(−u, 0, −w);// parallel to ZX	
l. Vertices[11] = VoxelPoint + new Vector3d(+u, 0, −w);// parallel to ZX	
3. Create Faces	
a. List<MeshFace>Faces = IntersectionTarget.Faces;	
b. Faces.AddFace(00, 01, 02, 03); //parallel to XY	
c. Faces.AddFace(04, 05, 06, 07); //parallel to YZ	
d. Faces.AddFace(08, 09, 10, 11); //parallel to ZX	
e. IntersectionTarget.Vertices.AddVertices(Vertices);	
f. IntersectionTarget.Faces.AddFaces(Faces);	
g. return IntersectionTarget;

The above algorithm is currently implemented such that it produces 12 quadrangular faces and thus 24 triangles. It will be therefore more efficient to implement the above function instead.

The function 1D_6_intersectionTarge described by the pseudo code in Algorithm 5 creates 1D mesh ensuring 6-connectivity. Note that this intersection target produces 12 triangles and that this number cannot be reduced, even if the other alternative for a 6-connected result were implemented (i.e. the outline faces of a voxel cube). This implies that if we want to create a better-connected raster with more voxels (6-connected), then we need to do more computation.

Algorithm 5 **1D_6_intersectionTarget.**

**Table:** 

**function:** 1D_6_intersectionTarget	
**input:** voxel size as Vector3d	
**output:** an intersection target for 1D input ensuring 6 connectivity as a mesh object as in the figure below	
1. Initialize	
a. Mesh IntersectionTarget = new Mesh();	
b. double u = (vSize.X / 2), v = (vSize.Y / 2), w = (vSize.Z / 2);	
c. Point3d[] Vertices = new Point3d[9]; Vertices[0] = VoxelPoint;	
2. Create vertices	
b. Vertices[1] = VoxelPoint − new Vector3d(+u, +v, +w);	
c. Vertices[2] = VoxelPoint − new Vector3d(−u, +v, +w);	
d. Vertices[3] = VoxelPoint − new Vector3d(−u, −v, +w);	
e. Vertices[4] = VoxelPoint − new Vector3d(+u, −v, +w);	
f. Vertices[5] = VoxelPoint + new Vector3d(−u, −v, +w);	
g. Vertices[6] = VoxelPoint + new Vector3d(+u, −v, +w);	
h. Vertices[7] = VoxelPoint + new Vector3d(+u, +v, +w);	
i. Vertices[8] = VoxelPoint + new Vector3d(−u, +v, +w);	
3. Create faces	
A. List<meshface>faces = intersectiontarget.faces;	
b. Faces.AddFace(0, 1, 2); Faces.AddFace(0, 2, 6);	
c. Faces.AddFace(0, 6, 5); Faces.AddFace(0, 5, 1);	
d. Faces.AddFace(0, 4, 1); Faces.AddFace(0, 5, 8);	
e. Faces.AddFace(0, 8, 7); Faces.AddFace(0, 7, 3);	
f. Faces.AddFace(0, 3, 4); Faces.AddFace(0, 4, 8);	
g. Faces.AddFace(0, 7, 6); Faces.AddFace(0, 2, 3);	
h. IntersectionTarget.Vertices.AddVertices(Vertices);	
i. IntersectionTarget.Faces.AddFaces(Faces);	
4. return IntersectionTarget;

The function BboxToVoxels, defined in Algorithm 6, can be used to full a bounding box with voxels.

Algorithm 6 **BBoxToVoxels.**

**Table:** 

**Fig. 6:**
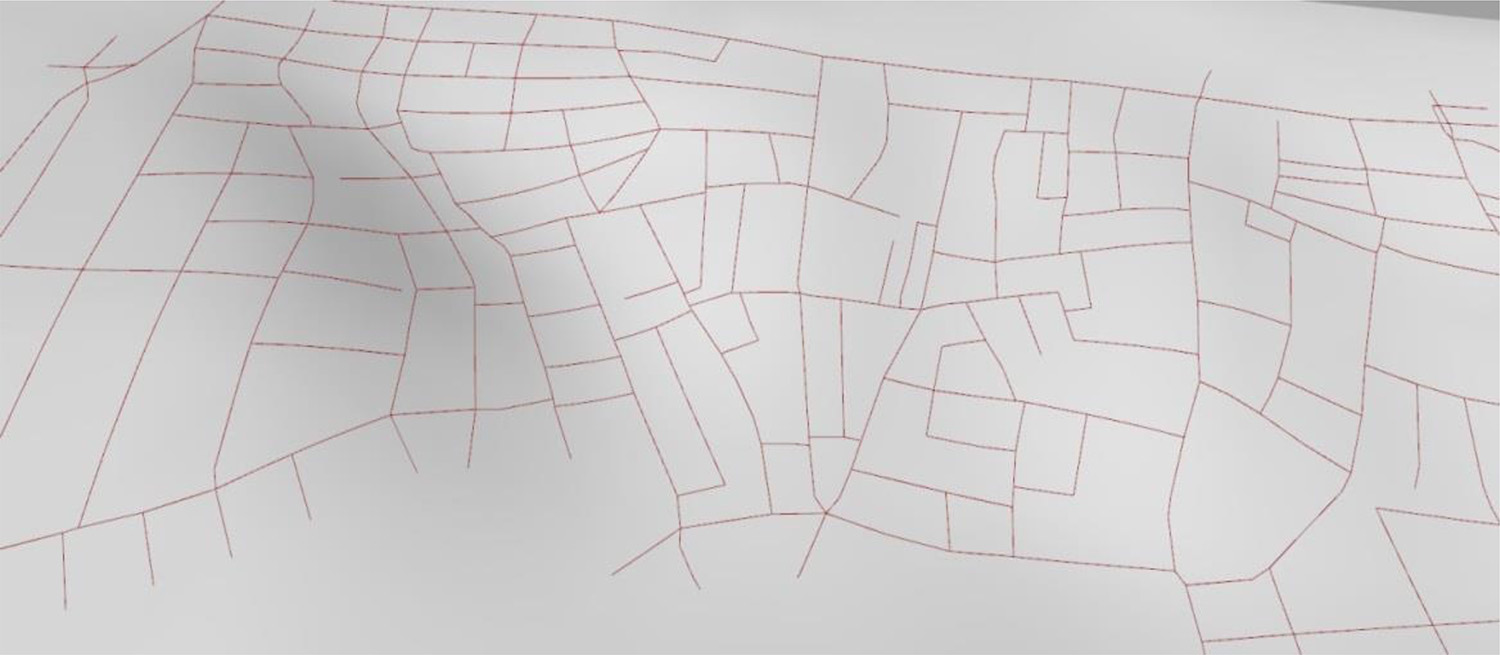
A set of curves extracted from OpenStreetMap representing a street network {Tarlabasi, Istanbul}.

**function: BBoxToVoxels**
**input**: bounding box adjusted to voxel size (ZBBox), voxel size as Vector3d (vSize)
**output**: an intersection target for 1D input ensuring 6 connectivity as a mesh object as in the figure below
1. Initialize
a. int i = 0, j = 0, k = 0;
b. $\text{int}\;\text{Imax}=\left[\left|ZBBox.Diagonal.X/Vsize.X\right|\right]$;
c.$\text{int}\;\text{Jmax}=\left[\left|ZBBox.Diagonal.Y/Vsize.Y\right|\right]$;
d. $\text{int}\;\text{Kmax}=\left[\left|ZBBox.Diagonal.Z/Vsize.Z\right|\right]$;
e. List<Point3d>VoxelPoints = new List<Point3d>();
2. Create voxels
a. for (k = 0; k < Kmax; k++) {for (j = 0; j < Jmax; j++){for (i = 0; i < Imax; i++)
i. Point3d RelPoint = new Point3d(i * vSize.X, j * vSize.Y, k * vSize.Z);
ii. RelPoint = RelPoint + new Vector3d(ZBBox.Min) + 0.5*VSize;
iii. VoxelPoints.Add(RelPoint);
3. Return VoxelPoints;

The voxelizeCurve algorithm has been applied to a dataset consisting of street centrelines of a neighbourhood in Istanbul called Tarlabasi, shown in Fig. 5, available on OpenStreetMaps.^6^ The results are shown in Fig. 6.

**Fig. 7:**
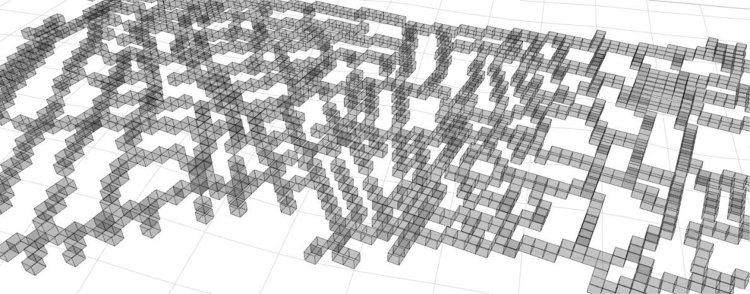
Voxelized curves (street centrelines) with 6-connectivity from Tarlabasi dataset with the resolution 10 × 10 × 10.

It is important to note that for 6-Connectivity the both of the targets mentioned in Table 1 guarantee 6 connectivity and in that sense are equally effective. However, the diagonal mesh target is only composed of 8 triangles whereas the cube outline target will be practically composed of 12 triangles. It is important to note that for solving intersections unambiguously an intersection between a ray or a half-line and a triangle should be found. This means that in practice the diagonal target will 12/8 = 1.5 times faster than a corresponding cube outline target. This is not the final conclusion on efficiency however. As for implementing Meta Inetrsection Targets, straight faces would be advantageous as they can be eventually replaced by finding intersections to half-planes (Fig. 7).

Note that every line in the voxelized curve network in Fig. 6 is 6-connected but the whole network is not. Ensuring such connectivity for the network would require extra measures and techniques.

### Surface voxelization (2D inputs, 1D targets)

Function voxelizeSurface is used for topological voxelization of 2D surfaces, which are represented as TIN or triangular polygon mesh objects. Note that other surfaces such as NURBS surfaces are also approximated as such for rendering purposes and so they can be the input of this method. The algorithm works by iterating over triangle faces of the surface in question by checking whether they intersect with the relevant intersection target of each voxel that could possibly be in resulted 3D raster. The points that possibly intersect with an intersection target are those whose distance to the input object is less than half of the length of the voxel size vector, i.e. a virtual vector comprised of the voxel size in X, Y and Z directions. Intersection between a connectivity target and the input surface is eventually determined by checking intersections between individual line segments and triangles. A standard algorithm for determining whether a triangle and a line intersect has been used for this purpose (see **Curve Voxelization (1D Inputs, 2D Targets)**).

Users can choose a level of connectivity (6 or 26), because of which a corresponding connectivity target will be chosen here to produce appropriate results. The pseudo code is described in Algorithm 6.

Algorithm 7 **voxelizeSurface.**

**Table:** 

**function:** voxelizeSurface
**input:** connectivity *int*, surfaces *mesh*, sx *double*, sy *double*, sz *double*
**output:** voxels *list*<*3D_points*>
1. for each surface in surfaces:
a. form a bounding box in ${\mathbb{R}}^{3}$ for curve;
b. define voxels as list of 3D points;
/* generate a bounding box equal or larger than the current bounding box in ${\mathbb{R}}^{3}$ (c.f., Algorithm 2) */
c. ZBBox = **RBBox_to_ZBBox**(RBBox, VSize);
d. define voxel size as vSize = [sx, sy, sz];
/* Pseudo code described by Algorithm 6*/
a. VoxelPoints = **BBoxToVoxels**(ZBBox,VSize);
/*voxel centre points closer than half of the length vSize to the surface which possibly intersect with the input surface;*/
e. near_voxel_points = VoxelPoints.FindAll(lambda| lambda.DistanceTo(Surface)<|VSize|/2
f. for each voxel_point in near_voxel_points
i. if (connectivity= =26)
/* Pseudo code described by Algorithm 8*/
1. intersectionTarget = **2D_26_intersectionTarget** (voxel_point, vSize);
ii. elseif (connectivity = = 6)
/* Pseudo code described by Algorithm 10*/
1. intersectionTarget = **2D_6_intersectionTarget** (voxel_point, vSize);
iii. else
1. throw exception and prompt: “other connectivity levels undefined”;
iv. if (**TriangleIntersectsLine**(intersectionTarget, surface))
1. voxels.add(voxel_point);
2. return voxels;

The function 2D_26_intersectionTarget described by the pseudo code in Algorithm 7 creates 2D mesh ensuring 26 connectivity.

Algorithm 8 **2D_26_intersectionTarget.**

**Table:** 

**function:** 2D_26_intersectionTarget	
**input:** voxel size as Vector3d	
**output:** an intersection target for 2D input ensuring 26 connectivity as a line array as in the figure below	
1. Initialize	
a. Line[] IntersecTarget = new Line[3];	
b. Point3d[] Vertices = new Point3d[6];	
c. double u = (vSize.X / 2), v = (vSize.Y / 2), w = (vSize.Z / 2);	
2. Create Vertices	
a. Vertices[0] = VoxelPoint + new Vector3d(+u, 0, 0);	
b. Vertices[1] = VoxelPoint + new Vector3d(0, +v, 0);	
c. Vertices[2] = VoxelPoint + new Vector3d(0, 0, +w);	
d. Vertices[3] = VoxelPoint + new Vector3d(−u, 0, 0);	
e. Vertices[4] = VoxelPoint + new Vector3d(0, −v, 0);	
f. Vertices[5] = VoxelPoint + new Vector3d(0, 0, −w);	
3. Create Edges	
a. for (int i = 0; i <= 2; i++)	
i. IntersecTarget[i] = new Line(Vertices[i], Vertices[i + 3]);	
b. return IntersectionTarget;

The function 2D_26_intersectionTargetDiagonal described by the pseudo code in Algorithm 8 creates 2D mesh ensuring 26 connectivity with diagonal target.

Algorithm 9 **2D_26_intersectionTargetDiagonal.**

**Table:** 

**function:** 2D_6_intersectionTargetDiagonal	
**input:** voxel size as Vector3d	
**output:** an intersection target for 2D input ensuring 6 connectivity as a line array as in the figure below	
1. Initialize	
a. Line[] IT = new Line[4];	
b. Point3d[] VX = new Point3d[8];	
c. double u = (vSize.X / 2), v = (vSize.Y / 2), w = (vSize.Z / 2);	
2. Create Vertices	
a. VX[0] = VoxelPoint + new Vector3d(+u, +v, +w);	
b. VX[1] = VoxelPoint + new Vector3d(−u, +v, +w);	
c. VX[2] = VoxelPoint + new Vector3d(−u, −v, +w);	
d. VX[3] = VoxelPoint + new Vector3d(+u, −v, +w);	
e. VX[4] = VoxelPoint + new Vector3d(−u, −v, −w);	
f. VX[5] = VoxelPoint + new Vector3d(+u, −v, −w);	
g. VX[6] = VoxelPoint + new Vector3d(+u, +v, −w);	
h. VX[7] = VoxelPoint + new Vector3d(−u, +v, −w);	
3. Create Edges	
a. IT[0] = new Line(VX[0], VX[4]);	
b. IT[1] = new Line(VX[1], VX[5]);	
c. IT[2] = new Line(VX[2], VX[6]);	
d. IT[3] = new Line(VX[3], VX[7]);	
e. return IT;

The function 2D_6_intersectionTarget described by the pseudo code in Algorithm 9 creates 2D mesh ensuring 6 connectivity with outline target.

Algorithm 10 **2D_6_intersectionTarget.**

**Table:** 

**function:** 2D_6_intersectionTarget	
**input:** voxel size as Vector3d	
**output:** an intersection target for 2D input ensuring 6 connectivity as a line array as in the figure below	
1. Initialize	
d. Line[] IT = new Line[12];	
e. Point3d[] VX = new Point3d[8];	
f. double u = (vSize.X / 2), v = (vSize.Y / 2), w = (vSize.Z / 2);	
2. Create Vertices	
i. VX[0] = VoxelPoint + new Vector3d(+u, +v, +w);	
j. VX[1] = VoxelPoint + new Vector3d(−u, +v, +w);	
k. VX[2] = VoxelPoint + new Vector3d(−u, −v, +w);	
l. VX[3] = VoxelPoint + new Vector3d(+u, −v, +w);	
m. VX[4] = VoxelPoint + new Vector3d(−u, −v, −w);	
n. VX[5] = VoxelPoint + new Vector3d(+u, −v, −w);	
o. VX[6] = VoxelPoint + new Vector3d(+u, +v, −w);	
p. VX[7] = VoxelPoint + new Vector3d(−u, +v, −w);	
3. Create Edges	
f. IT[00] = new Line(VX[0], VX[1]);	
g. IT[01] = new Line(VX[1], VX[2]);	
h. IT[02] = new Line(VX[2], VX[3]);	
i. IT[03] = new Line(VX[3], VX[0]);	
j. IT[04] = new Line(VX[0], VX[6]);	
k. IT[05] = new Line(VX[6], VX[5]);	
l. IT[06] = new Line(VX[5], VX[4]);	
m. IT[07] = new Line(VX[4], VX[7]);	
n. IT[08] = new Line(VX[5], VX[3]);	
o. IT[09] = new Line(VX[4], VX[2]);	
p. IT[10] = new Line(VX[1], VX[7]);	
q. IT[11] = new Line(VX[6], VX[7]);	
r. return IT;

Similar to the case of mesh targets for curve inputs, it is notable that each diagonal line target is only composed of 8 lines (which can also be made with 4 lines) whereas the outline target composed of cube edges is consisted of 12 lines. This means that the diagonal target is 12/8 = 1.5 times faster for computing voxels in the output compared to the cube outline edges. This is because for each voxel to be included in the output in the case of cube outline edges there must be 12 intersections solved in the worst case whereas the maximum number of line triangle intersections in the case of diagonal targets is 8. However, it might be advantageous to work with the cube edges because in that case 2D scan conversions parallel to X, Y, or Z axes might be combined (in a Meta Intersection Target) to find out voxels in the output. This is important because 2D scan conversion is a standard procedure for graphical processors that pixelate vector inputs for visualization on 2D digital monitors. Treating this matter in depth falls outside of the scope of this paper though (Fig. 8, Fig. 9).

### Notes on implementation and application

Our implementation of point cloud voxelization algorithm and curve voxelization algorithm are using rhinocommon.dll^7^ that is a library provided by McNeel,^8^ the vendor of Rhino3D.^9^ The dependencies however are mostly because of using geometric type definitions such as Point3D, Line and Mesh, which can be replaced straightforwardly. An example code without such dependencies is the surface voxelization code provided in the repository. Codes dependant on Rhinocommon can be complied and tested in an environment such as Grasshopper3D^10^ also developed by McNeel.

As can be seen in curve and surface voxelization algorithms, we are currently forming a 3D grid full of voxels for a bounding box of the input object. This can be problematic in case this bounding box is big. In case of surfaces, it is more efficient to iterate over individual triangle faces to avoid this problem. Similarly, it will be more efficient to iterate over individual line segments in a polyline approximating the input curve compared to iterating over curve objects directly.^11^ This, however, means that there will be many duplicate voxels in the raster output. To avoid this problem, a 3D raster data model similar to the model implemented in the voxelization algorithm for point clouds needs to be implemented to keep track of voxels already created and to ensure there will not be duplicates. Such a 3D raster data model will be possibly very space efficient as it should only store one bit per voxel. Such an implementation then will bring other advantages such as the ease of Boolean operations on multiple 3D raster objects such as Boolean operations (union, intersection, etc.) and mathematical morphology operations (erosion, dilation, gradient, etc.) as it will only involve bitwise operations, provided that every two 3D raster models are defined in reference to the same bounding box so that their ${\mathbb{R}}^{3}$ representations are compatible. Then such a requirement imposes another requirement that regards the existence of many zeros in any such 3D raster data model. Consider a house model voxelized in reference to a bounding box over the whole neighbourhood or city. It is obvious that most of the voxels represented in the 3D array of bits are zero. This suggests implementation of a data structure like sparse matrix so that such 3D raster models can be compressed in memory.

What is interesting about a binary representation of a voxel collection (as 3D raster) is that the same way 2D raster data models (2D images) can be processed and analyzed, 3D raster models (3D images if you like) can be processed and analyzed. What is common in both cases is the binary representation of coloured picture elements (pixels) or coloured volume elements (voxels). In short, we can enter a completely new world of possibilities inspired by digital image processing, merely by representing voxel grids as Boolean tensors (3D matrices).

## Figures and Tables

**Fig. 8:**
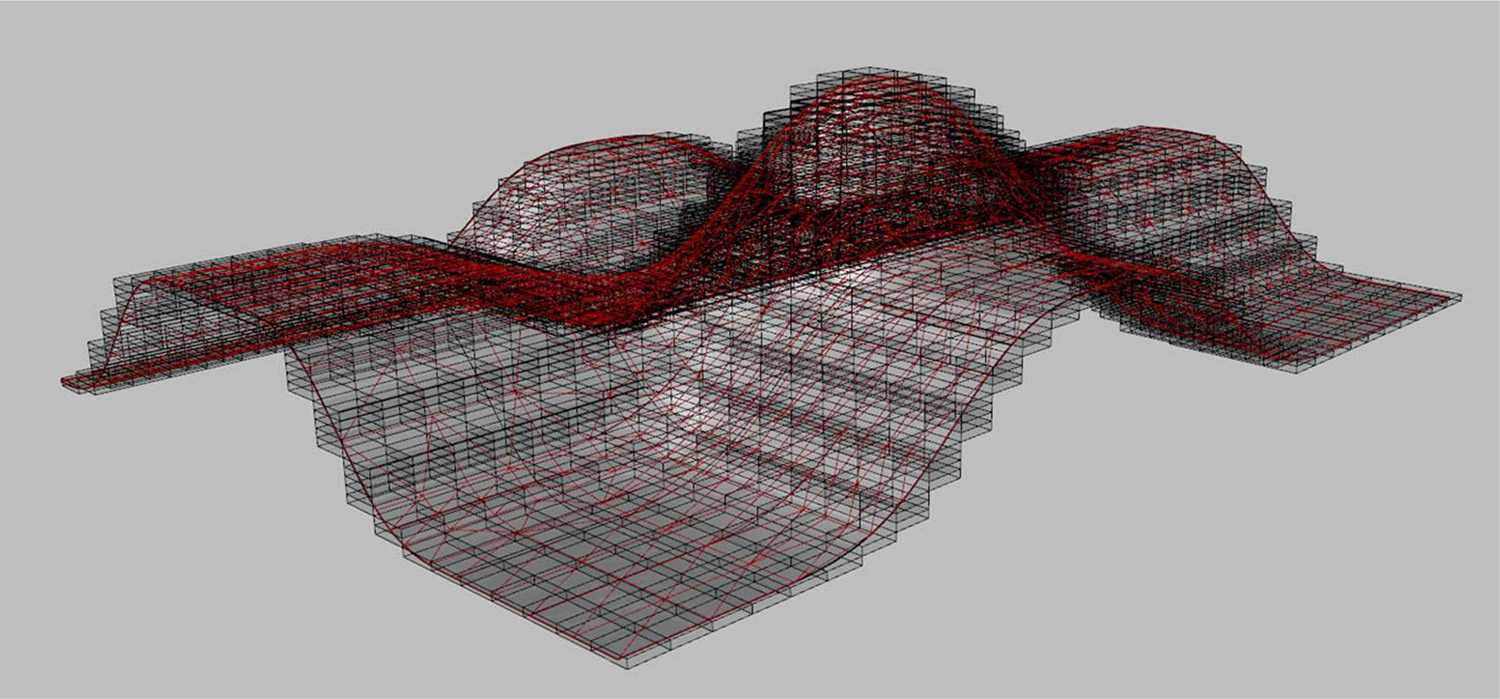
A surface (triangular polygon mesh) voxelized with connectivity level 26.

**Fig. 9:**
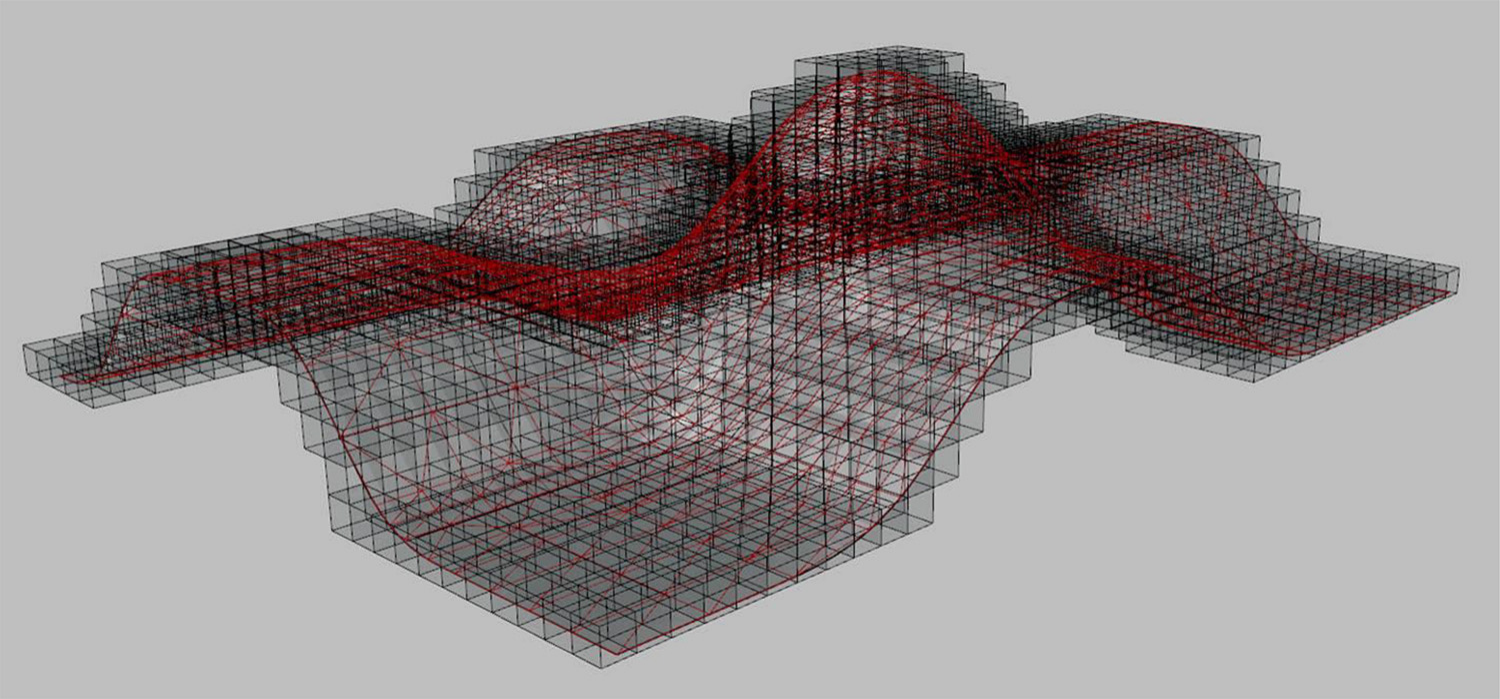
A surface (triangular polygon mesh) voxelized with connectivity level 6.

**Table 1:** Intersection targets for topological voxelization reproduced after [1].

	Output connectivity	
	6-connected	26-connected
1D (curve) inputs		
	
2D (surface) inputs		
	

**Table 2:** Duality of features in 1D space.

Primal feature	Dual feature
0D vertex (e.g. a point)	1D edge
1D edge (e.g. a line segment)	0D vertex

**Table 3:** Duality of features in 2D space.

Primal feature	Dual feature
0D vertex (e.g. a point)	2D face
1D edge (e.g. a line segment)	1D edge
2D face (e.g. a triangle or a pixel)	0D vertex

**Table 4:** Duality of features in 3D space.

Primal feature	Dual feature
0D vertex (e.g. a point)	3D body
1D edge (e.g. a line segment)	2D face
2D face (e.g. a triangle or a pixel)	1D edge
3D body (e.g. a tetrahedron or a voxel)	0D vertex

## Footnotes

1 Weisstein, Eric W. "von Neumann Neighbourhood." From *MathWorld* – A Wolfram Web Resource. http://mathworld.wolfram.com/vonNeumannNeighborhood.html.

2 Weisstein, Eric W. "Moore Neighbourhood." From *MathWorld* – A Wolfram Web Resource. http://mathworld.wolfram.com/MooreNeighborhood.html.

3 Weisstein, Eric W. "Cellular Automaton." From *MathWorld* – A Wolfram Web Resource. http://mathworld.wolfram.com/CellularAutomaton.html.

4 We have not included an algorithm for this case, as it seemed quite straightforward.

5 Note that in our current implementation of the curve voxelization algorithm we are using the Curve-Mesh intersection algorithm from Rhinocommon. This algorithm can be easily rewritten using the line-triangle intersection algorithm (Algorithm 10), provided that the input curve is approximated as a polyline composed of line segments. This is a normal procedure as such approximation is needed for all kinds of curves such as conic sections and NURBS prior to rendering.

6 https://www.openstreetmap.org.

7 http://4.rhino3d.com/5/rhinocommon/. See also: https://github.com/mcneel/rhinocommon.

8 http://www.en.na.mcneel.com/.

9 https://www.rhino3d.com/.

10 http://www.grasshopper3d.com/.

11 This is not the case in our current implementation now. We are iterating over ‘generic curve objects’ and use a function of Rhinocommon to intersect a curve and a mesh. However, deep inside, this function is also based on intersecting line segments and triangles. Note that free-form curve objects such as NURBS curves are rendered as polylines with fine line segments.
