# Supplementary material for: Voxelization algorithms for geospatial applications: Computational methods for voxelating spatial datasets of 3D city models containing 3D surface, curve and point data models
Source: MethodsX. 2016 Jan 13;3:69–86. doi: 10.1016/j.mex.2016.01.001 (PMC4929271; doi:10.1016/j.mex.2016.01.001)
Supplement: Supplementary file 1 [file mmc1.docx]

## **Additional Information and Supplementary Materials**

### Rasterizing CAD models

Rasterization of a building model (Bentley building) can be done using the C# version of the code in Rhinoceros; as shown in the following images (see our exemplary immplementation of the C# source for Rhino3D and Grasshopper 3D here: <https://github.com/NLeSC/geospatial-voxels/tree/master/software/voxelGen/voxelizationTools_Rhino_and_GH>):


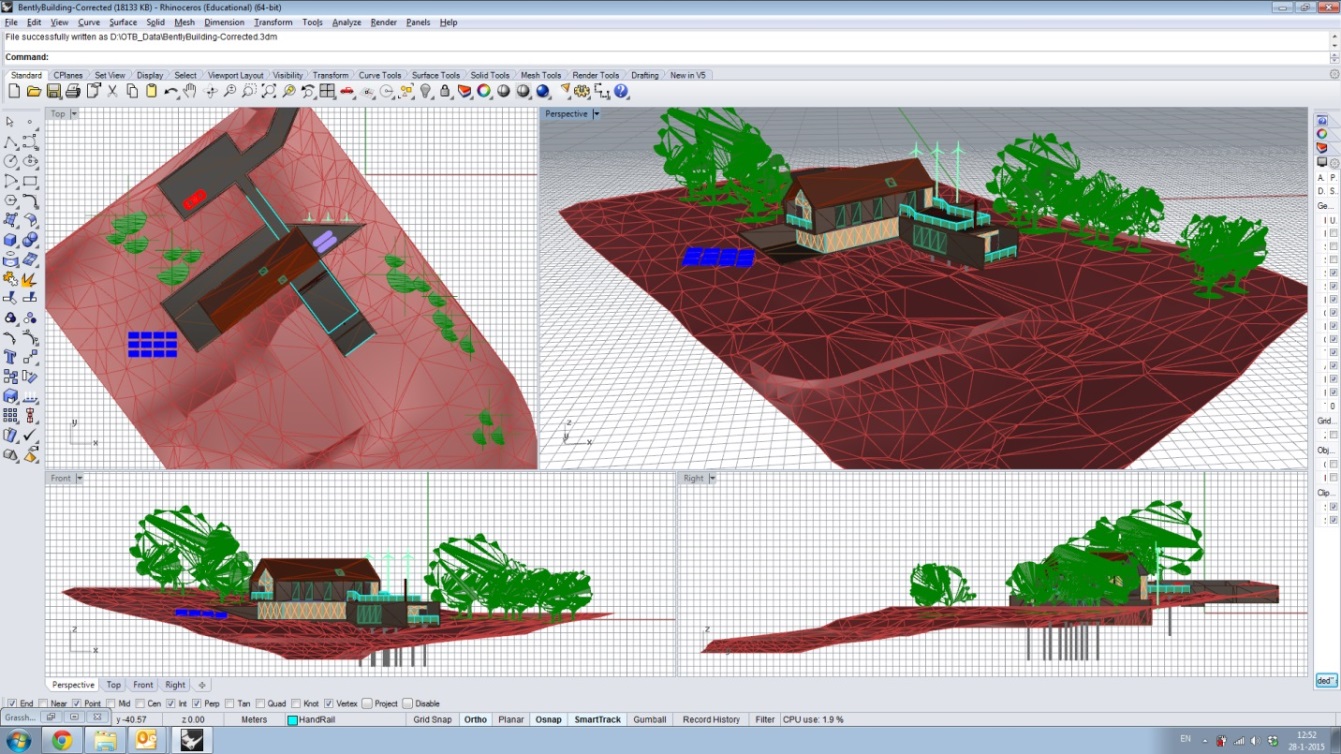


Figure 10:An IFC building mode provided by Bentley Systems, imported as OBJ generated from original BIM model, corrected and coloured to represent objects of different semantics


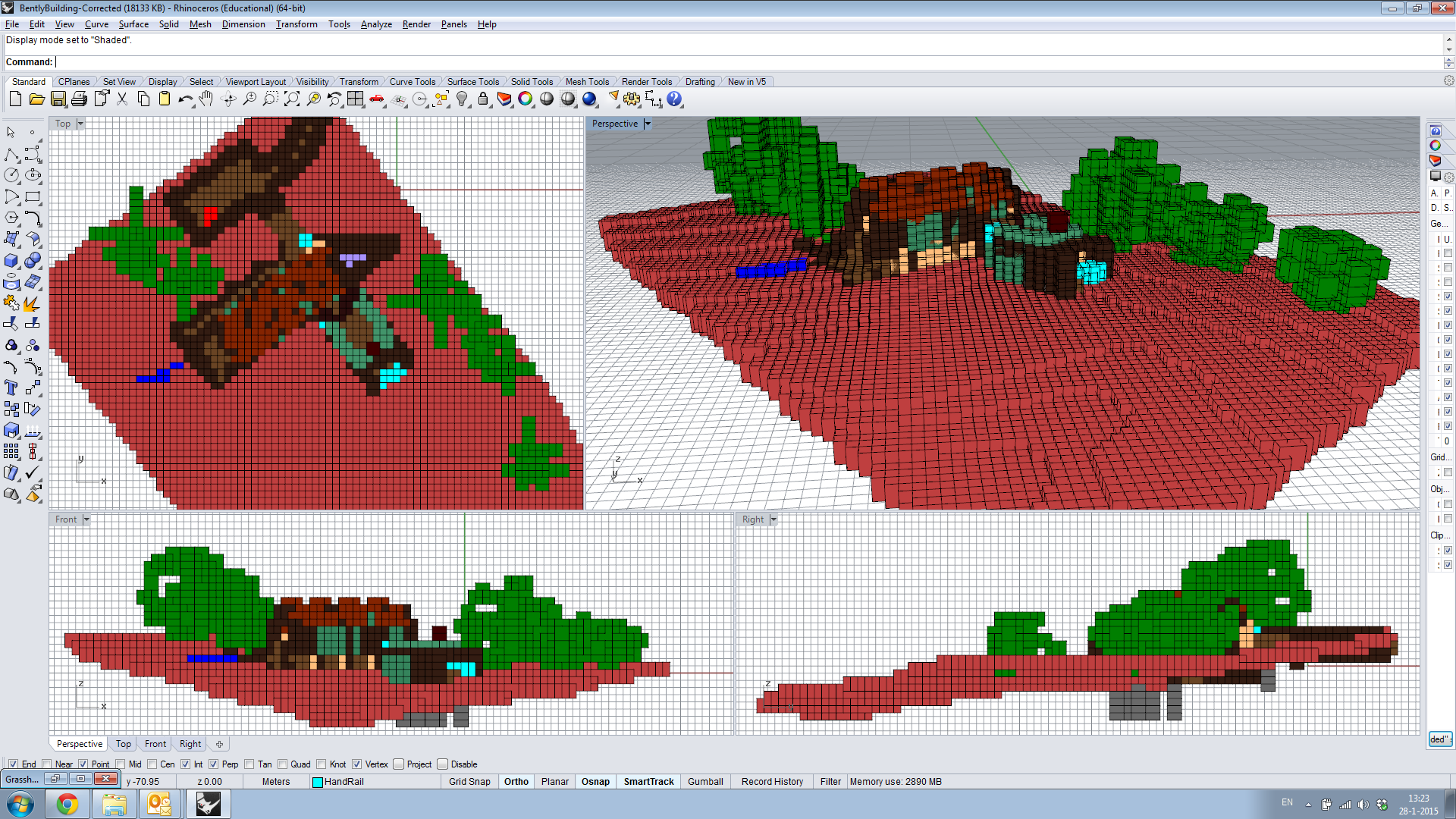


Figure 11: Topological Rasterization with Semantics from a BIM model [0.5 by 0.5 by 0.5 M]


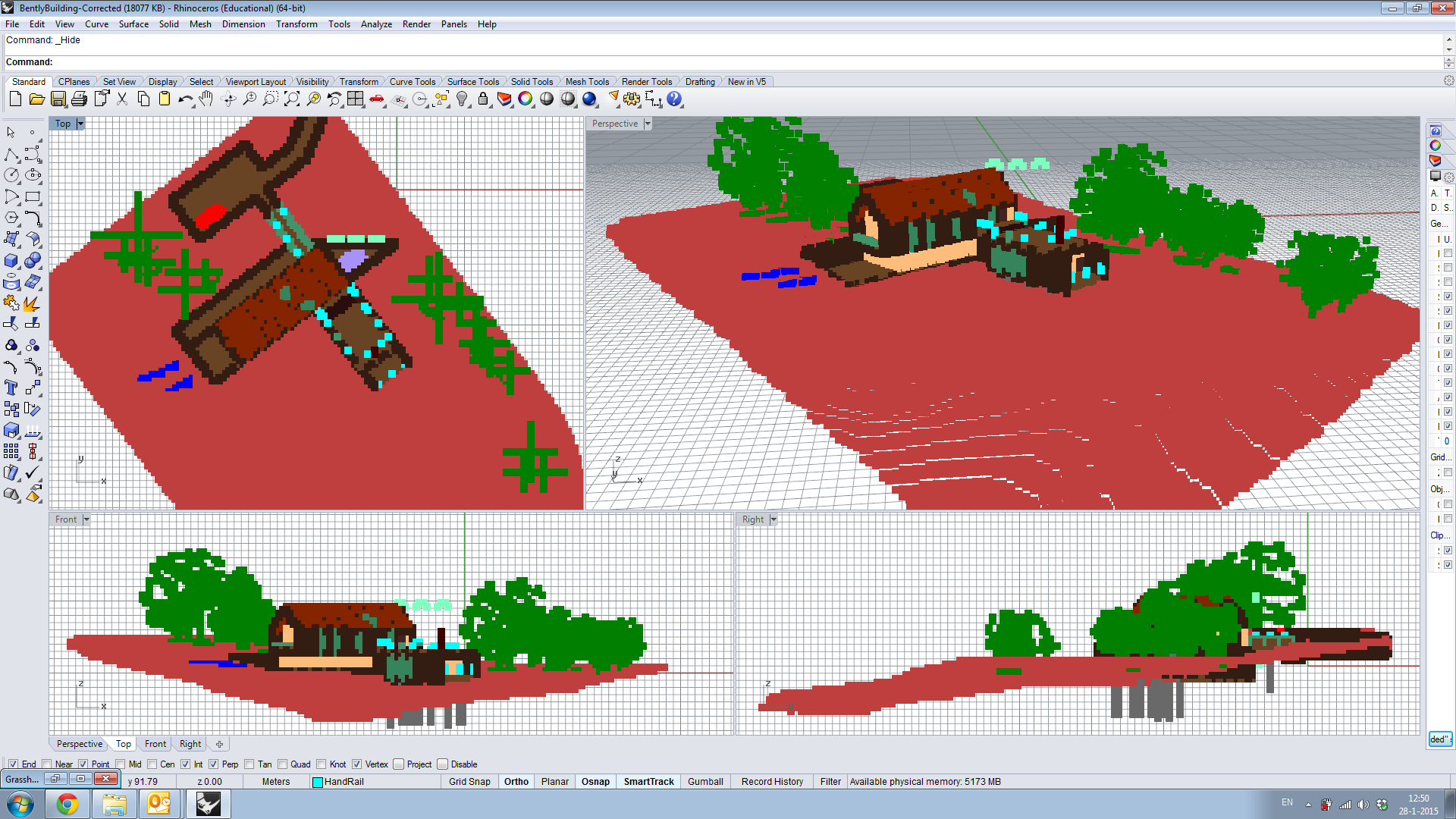


Figure 12: Topological Rasterization with Semantics from a BIM model [0.4 by 0.4 by 0.4 M]


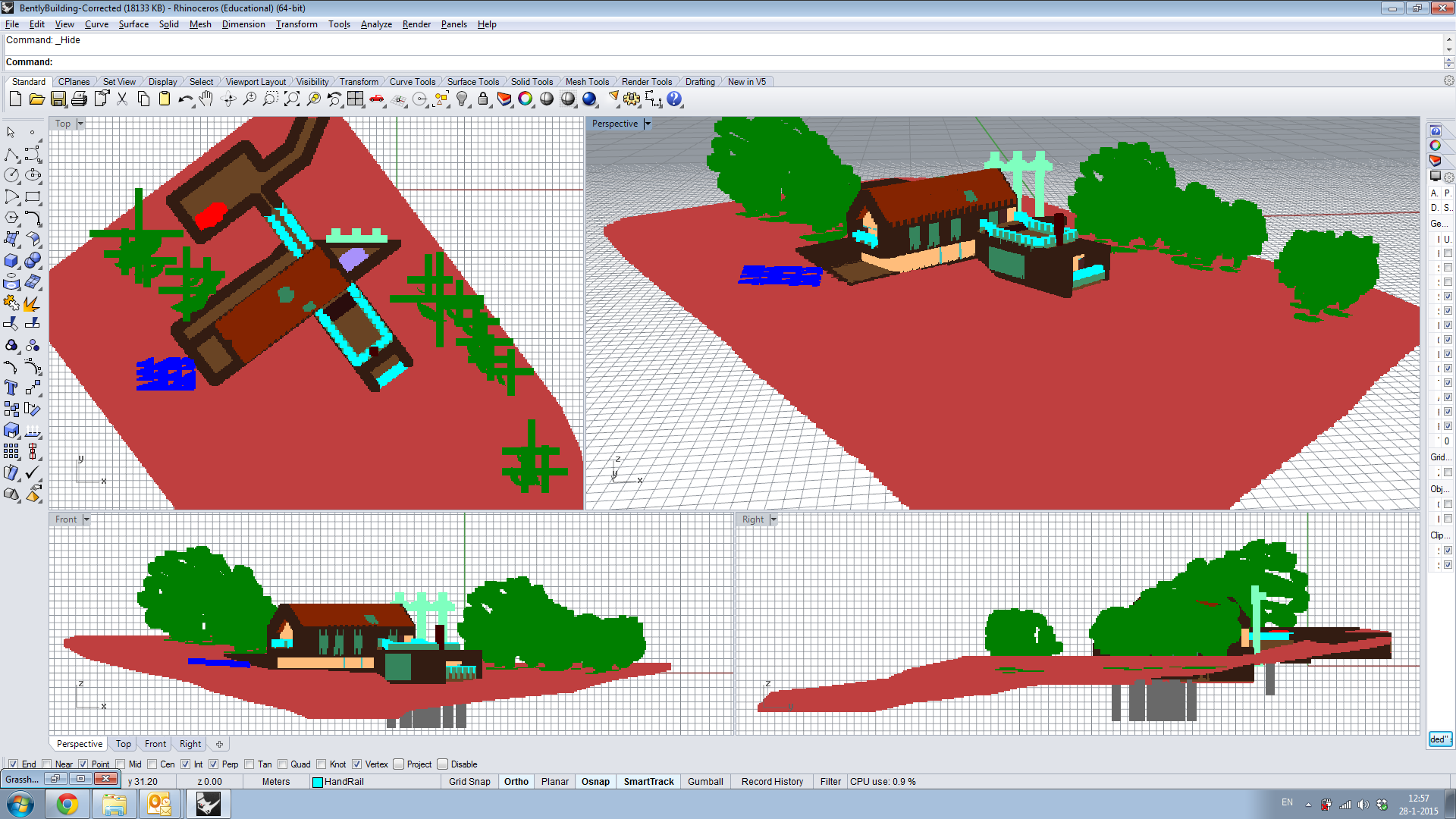


Figure 13: Topological Rasterization with Semantics from a BIM model [0.1 by 0.1 by 0.1 M]

### Rasterizing CityGML LOD2 models (GIS)

In voxelizing CityGML files the main issues are defining a proper schema both for raster 3D and voxels in order to keep semantic information such as the ones modelled in a CityGML. Both of these issues and also the matter of ensuring uniqueness of voxels (avoiding duplicates when joining multiple voxel collections) requires further investigation and implementation of measures such as a 3D binary array to keep track of visited voxels as implemented in our point cloud voxelization algorithm.The following images are test results of the last version of the TUD_voxelizer code in C#, presented in the aforementioned repository. Visualizations are done in CloudCompare.


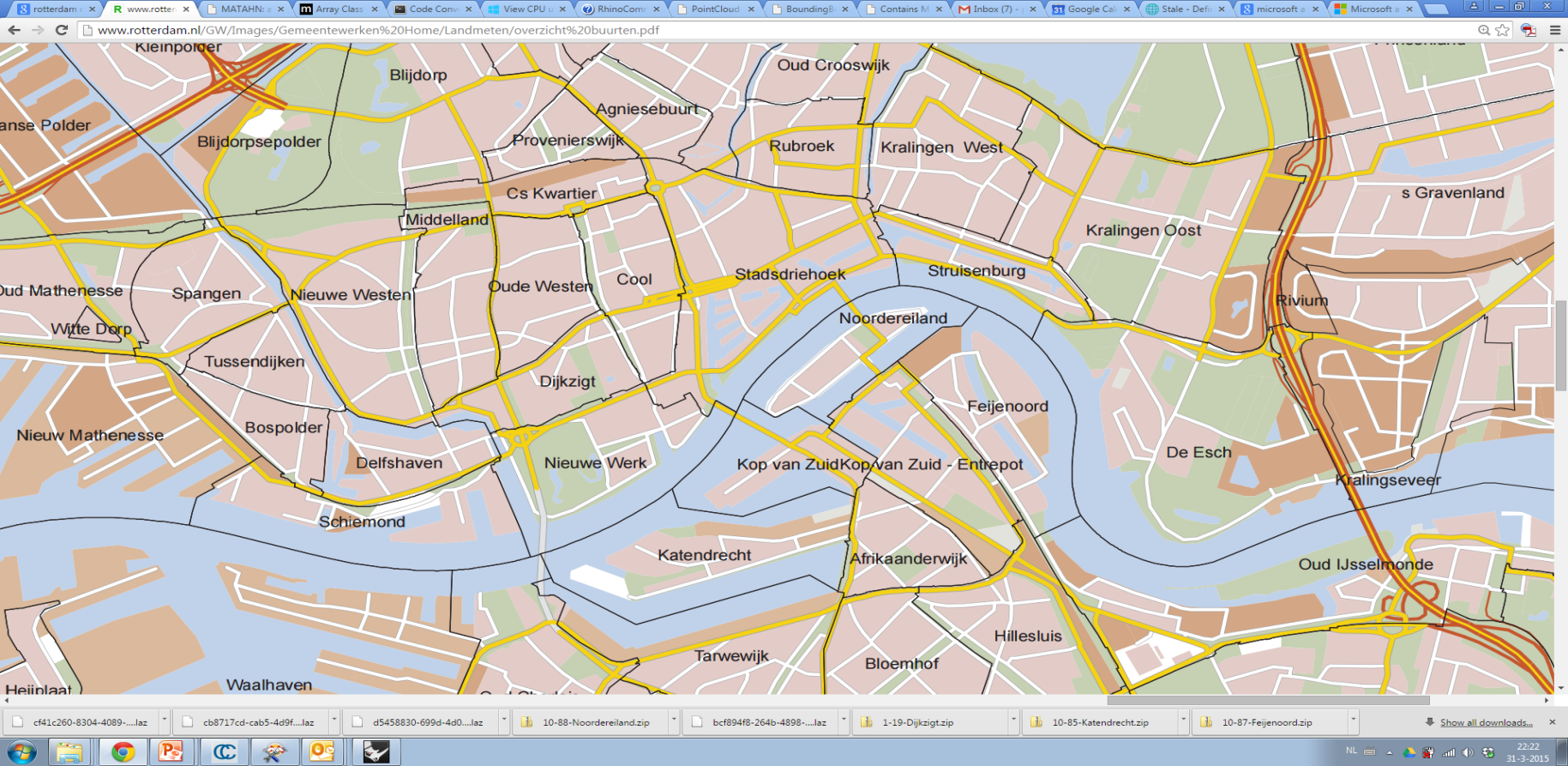


Figure 14: The area voxelized using CItyGML models provided by the municipality of Rotterdam. See the data sets and a sample voxelated result at: <https://github.com/NLeSC/geospatial-voxels/tree/master/software/voxelGen/data>


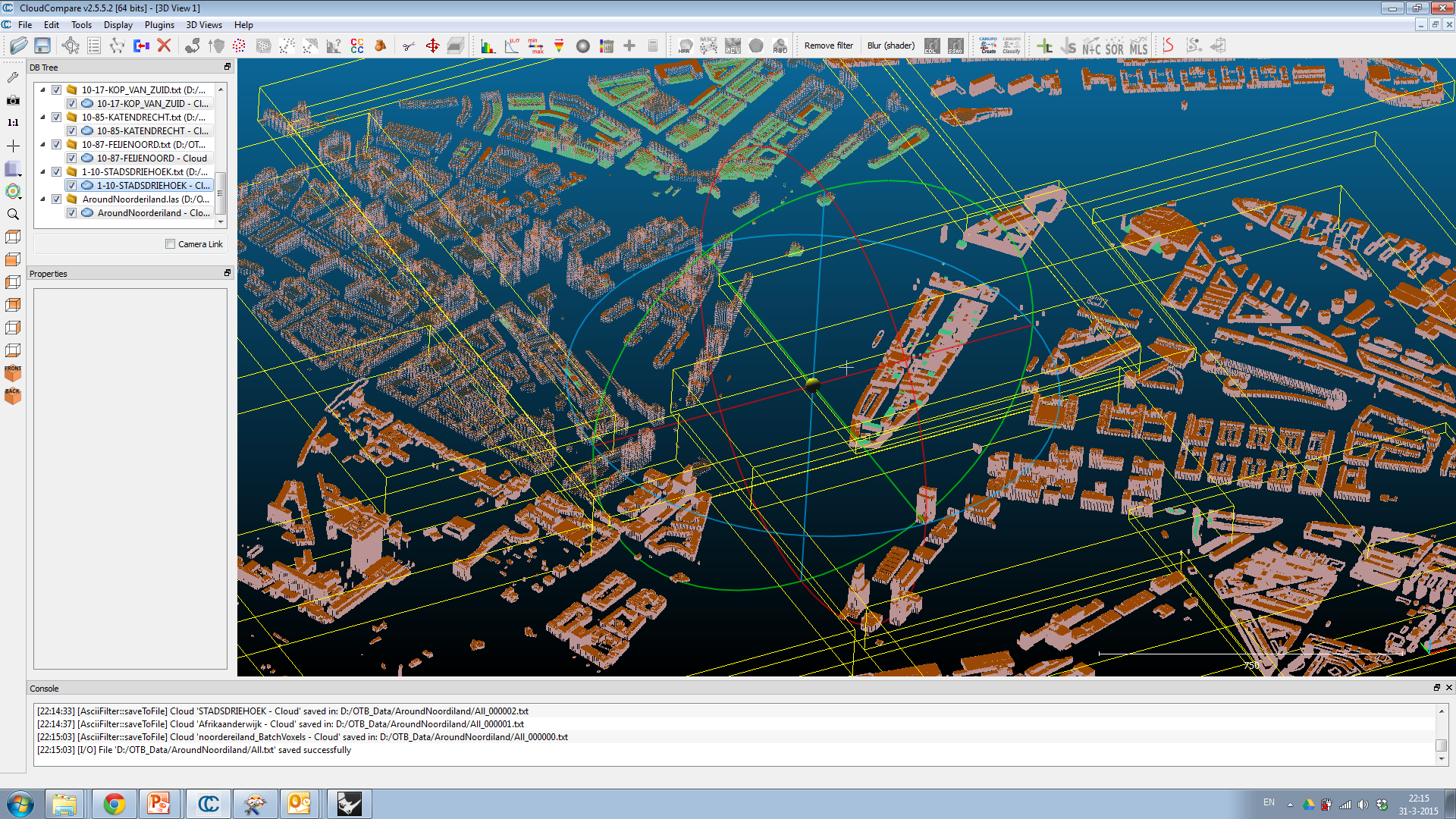


Figure 15: Rotterdam CityGML, Noordereiland, and neighbourhoods around it; voxelized in different resolutions, zoomed out.


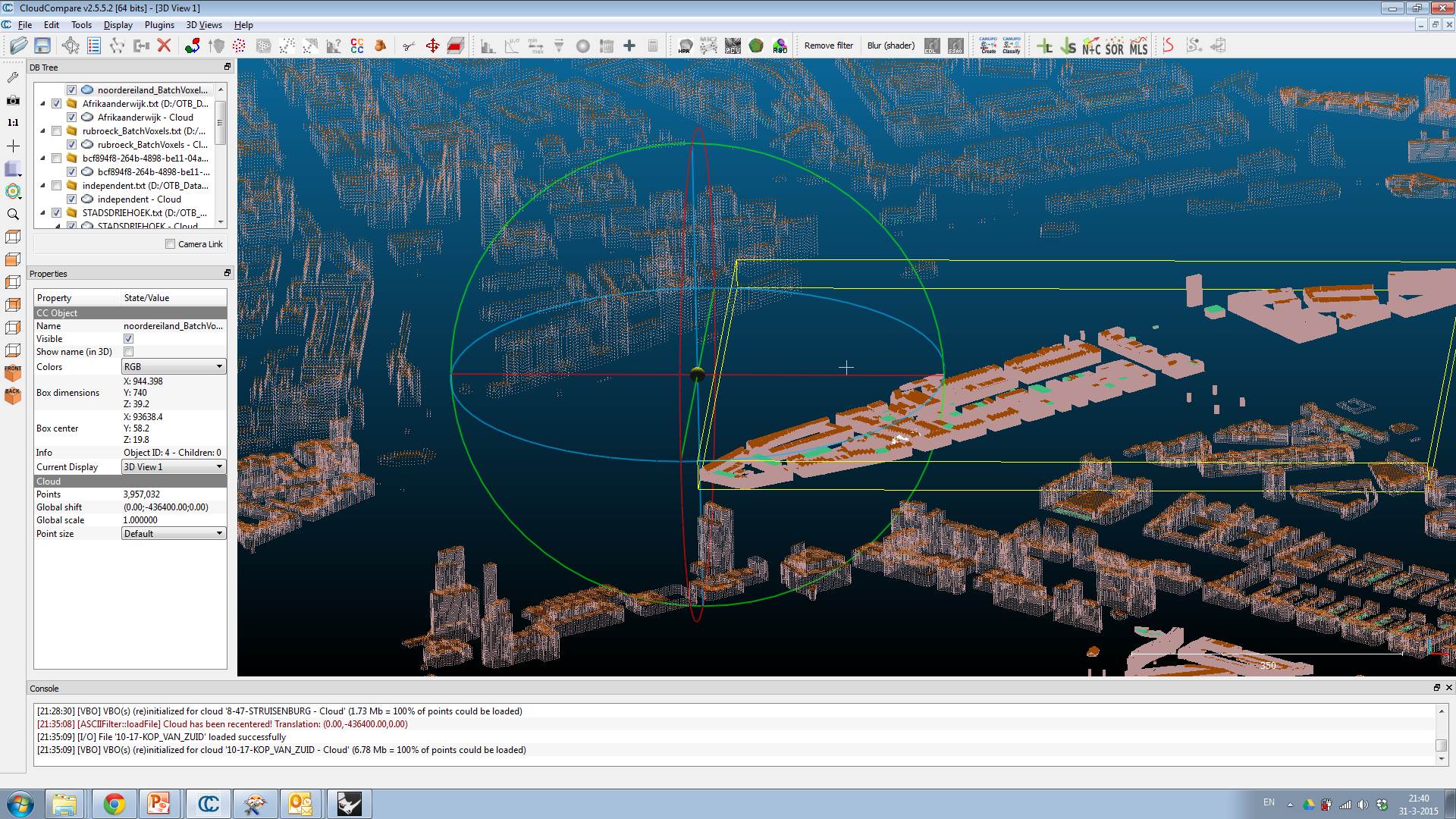


Figure 16: Rotterdam CityGML, Noordereiland and neighbourhoods around it; Noordereiland selected


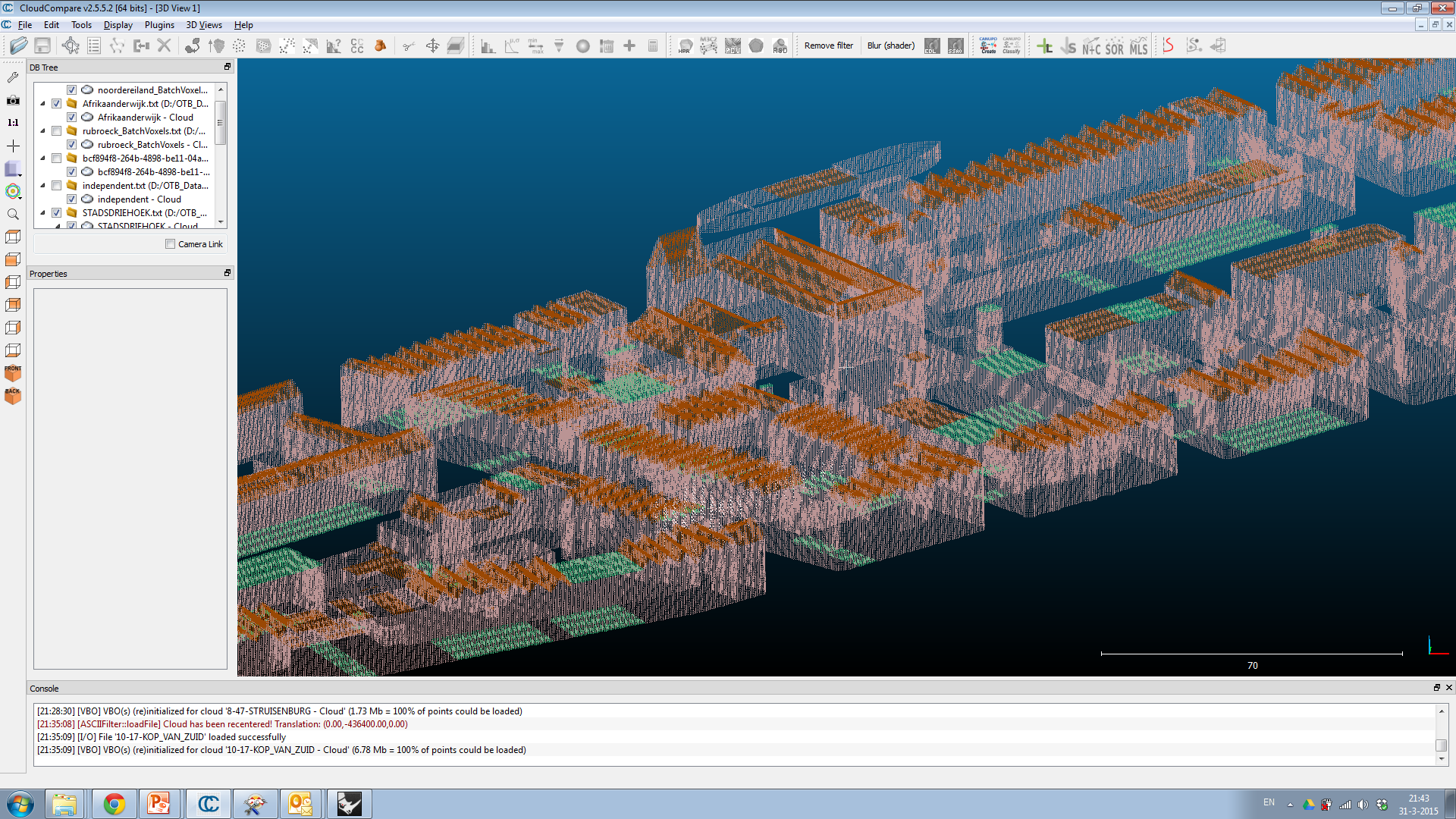


Figure 17: Rotterdam CityGML, Noordereiland, 0.2x0.2x0.2; zoomed in.

## **Background and Literature Review**

In this section, we briefly compare (qualitatively)our developments with other 3D rasterization algorithms for curves and surfaces. Since the geospatial objectsare commonly represented by multiple surfaces, the review is specifically on voxelizing surfaces.Use of volumetric primitives (cone, cylinder, sphere) to represent buildings, bridges, e.g. in BIM (Building information modelling)are excluded. Research discussing voxelisation of such primitives can be found in (Fang, S. & Chen, H., 2000) or(Jones, M. W., & Satherley, R., 2000).

Two general approaches can be distinguished in voxelisation: object rasterization (e.g. Topological Voxelization) and scan-conversion (e.g. 3D Scan Conversion) in the body of existing methods. Object rasterization concentrates on the object of interest following two steps: boundary rasterization (and possibly interior filling). The scan conversion focuses on the bounding box of the objects and decides that in a given raster volume which voxels get what kind of value. Most of the presented methods in thisgroupare based on extensions of the well-known scan-conversion method, which is well studied and commonly used in the field of 2D computer graphics.After an initial investigation of methods in a rather historical order(Table 5), we chose the following methods to implement and compared them according to our quality criteria:

- 3D Scan Conversion (Kaufman, 1987): i.e. extended 2D scan conversion into 3D. This method is very efficient but 6-Connectivity cannot be reached, therefore 26-separability not assured, difficult to generalize the method to different geometric primitives.
- Topological Voxelization (Laine, 2013) NVIDIA: based on the concept of connectivity target, very elegant mathematically; which can be efficiently implemented. The topological approach focuses on matters of Boolean nature such as connectivity, separability, intersections more explicitly compared to former geometrical approaches.

If there is any interest in doing the same comparison, we refer the reader to our implementation of both methods at:<https://github.com/NLeSC/geospatial-voxels/tree/master/software/voxelGen>. However, giving full account of our implementation of 3D scan conversion falls out of the scope of this paper.

| Year | 1987 | 1995 | 1998 | 2003 | 2010 | 2013 |
| --- | --- | --- | --- | --- | --- | --- |
| Authors | (Kaufman, A. & Shimony, E., 1987) | (Cohen-OR D., Kaufman A., 1995) | (Huang, J, Yage R, Filippov V and Kurzion Y, 1998) | (Varadhan G., Krishnan S., Kim Y.J., Diggavi S. Manocha D., n.d.) | (Schwarz M., Seidel H. P., 2010) | (Laine, 2013) |
| Method | 3D Scan Conversion (lines) | 3D Scan Conversion (surfaces) | Distance Based (Spheres) | Max-Norm distance | parallel voxelization using GPUs | Topological Voxelization |
| Explicit topology control? | Yes only for 26-connectivity/6 separation | Yes only for 26 connectivity/6 separation | Yes | N/A | yes | yes |
| Notes | 6-connected results cannot be produced | 6-connected results cannot be produced, multiple algorithms for different types of surfaces | is not minimal and it is sensitive to tessellation | produces a ‘cover’ voxel set, can be eventually used for improving efficiency of other methods | using an unnecessarily large target results in voxels that are not required for 6-separation, thus not minimal | Elegant method that comes with mathematical proofs for topological properties; algorithm not clearly defined. |

Table 5:aqualitative comparison of voxelization algorithms

# Works Cited

Arroyu Ohori, K., H. Ledoux, M. Meijer, 2012. Validation and automatic repair of planar partitions using a constrained triangulation. *Photogrammetrie-Fernerkundung-Geoinformation,* 5(10), pp. 613-630.

Cohen-OR D., Kaufman A., 1995. Fundamentals of surface voxelization. *Graph. Models Image Process,* 57(6), pp. 453-461.

Egenhofer, M.J. & Hering, J.R, 1990. A mathematical framework for the definition of topological relations. *Proceedings of the Fourth International Symposium on SDH*, pp. 803-813.

Fang, S. & Chen, H., 2000. Hardware accelerated voxelisation. *Computer & Graphics,* 24(3), pp. 433-442.

Huang, J, Yage R, Filippov V and Kurzion Y, 1998. An Accmate Method for Voxetiting Polygon Meshes. In: *IEEE Symposium on Volume Visualization (Cat. No.989EX300).* s.l.:IEEE, p. 119–126.

Jones, M. W., & Satherley, R., 2000. Voxelisation: Modelling for volume graphic. *Vision, Modeling, and Visualisation*, p. 8.

Kaufman, A. & Shimony, E., 1987. *3D scan-conversion algorithms for voxel-based graphics..* New York, ACM Press, p. 45–75.

Laine, S., 2013. A Topological Approach to Voxelization. *Computer Graphics Forum,* 32(4), p. 77–86.

Lee, J., Li, K-J., Zlatanova, S., Kolbe, T.H., Nagel, C., & Becker, T., 2014. *OGC. IndoorGML.* [Online]
Available at: http://docs.opengeospatial.org/is/14-005r3/14-005r3.html
[Accessed 12 December 2015].

Lee, J., 2001. *3D Data Model for Representing Topological Relations of Urban Features.* San Diego, CA, s.n.

Louwsma, J., Zlatanova, S. , van Lammeren, R. & van Oosterom, P., 2006. Specifications and implementations of constraints in GIS. *Geoinformatica,* 10(4), pp. 62-79.

Munkres, J., 1984. *Elements of Algebraic Topology.* Menlo Park, CA: Addison-Wesley.

Pigot, S., 1991. *Topological models for 3d spatial information systems.* s.l., ASPRS American Society of Photogrammetry and Remote Sensing, pp. 368-368.

Schwarz M., Seidel H. P., 2010. Fast paralell surface and solid voxelization on GPUs. *ACM trans. Graph. ,* 29(6), pp. 179:1-179:10.

Varadhan G., Krishnan S., Kim Y.J., Diggavi S. Manocha D., n.d. *Effeicient max-norm distance computation and reliable voxelization.* s.l., s.n., pp. 116-126.

Zlatanova, 2000. On 3D topological relationships. *The 11th International workshop on Database and Expert System Applications (DEXA 2000)*, 6-8 September, pp. 913-919.
